# Changes of the coronal lumbar-pelvic-femoral alignment after conversion total hip arthroplasty in patients with unilateral ankylosed hip

**DOI:** 10.1038/s41598-023-32672-8

**Published:** 2023-04-04

**Authors:** Takaomi Kobayashi, Tadatsugu Morimoto, Hirohito Hirata, Tomohito Yoshihara, Masatsugu Tsukamoto, Motoki Sonohata, Masaaki Mawatari

**Affiliations:** 1 Department of Orthopaedic Surgery, Faculty of Medicine, Saga University, 5-1-1 Nabeshima, Saga, 849-8501 Japan; 2 Department of Preventive Medicine, Faculty of Medicine, Saga University, 5-1-1 Nabeshima, Saga, 849-8501 Japan; 3 Department of Orthopaedic Surgery, Japan Community Health Care Organization (JCHO) Saga Central Hospital, 3-8-1 Hyogo Minami, Saga, 849-8522 Japan

**Keywords:** Medical research, Outcomes research

## Abstract

To elucidate the changes in coronal lumbar-pelvic-femoral alignment after conversion total hip arthroplasty (THA) in patients with unilateral ankylosed hip. A retrospective radiologic study of 48 patients (48 hips) with unilateral hip arthrodesis who underwent conversion THA was conducted. Cobb’s angle of lumbar scoliosis (LS), the pelvic obliquity (PO) angle, and the hip adduction angle (HAA) on standing anterior–posterior spine-pelvis-hip radiographs were measured before and after THA. The differences of LS, PO, and HAA before and after THA were defined as ΔLS, ΔPO, and ΔHAA, respectively. A paired samples t-test or the Wilcoxon signed-rank test were used to compare the absolute values of the LS, PO, and HAA between preoperative and postoperative groups. The Pearson’s correlation coefficient (r) or Spearman’s correlation coefficient (ρ) was calculated to assess the relationship between ΔLS, ΔPO, and ΔHAA and possible associated factors. Significant differences were found in the preoperative LS (mean, 10.8° vs. 8.2°, p = 0.004), PO (median, 6.8° vs. 2.0°, p < 0.001), and HAA (median, 10.0° vs. 6.0°, p = 0.003). ΔLS was correlated with the preoperative LS (ρ =  − 0.621, p < 0.001), PO (ρ =  − 0.580, p < 0.001), and HAA (ρ =  − 0.467, p < 0.001). ΔPO was correlated with the preoperative LS (r =  − 0.596, p < 0.001), PO (ρ =  − 0.892, p < 0.001), and HAA (ρ =  − 0.728, p < 0.001). ΔHAA was correlated with the preoperative LS (r =  − 0.583, p < 0.001), PO (ρ =  − 0.751, p < 0.001), and HAA (ρ =  − 0.824, p < 0.001). LS, PO, and HAA were significantly improved after conversion THA. Greater improvement in LS, PO, and HAA can be expected in patients with larger preoperative LS, PO, and HAA values.

## Introduction

An ankylosed hip is a painless joint due to complete immobilization with a patient satisfaction rate of 69–100%^1–7^. In contrast, an inadequate hip position frequently causes compensatory scoliosis (i.e., ‘*windswept hip-spine deformity*’^1^) and/or pain in the adjacent joints (e.g., lower back, ipsilateral knee, contralateral knee, and contralateral hip^2^) with time. These could be indications for conversion total hip arthroplasty (THA)^2–12^. In fact, conversion of hip arthrodesis to arthroplasty was found to result in pain relief in the adjacent joints in 66–100% of cases and patient satisfaction in 63–100% of cases^8–12^. Possible reasons for these improvements include the improvement of hip joint mobility and lumbosacral spine alignment and their associated reduction in load on the adjacent joints. However, the changes in coronal lumbar-pelvic-femoral alignment due to conversion of hip arthrodesis to movable arthroplasty remain unclear. This study therefore aimed to elucidate the changes in coronal lumbar-pelvic-femoral alignment after conversion THA in patients with unilateral ankylosed hip.

## Methods

### Study design

The Institutional Review Board of the University of Saga at Saga city approved this study and informed consent was obtained from all participants (Registration No.: 2015-12-13). This study also adhered to the principles of the Declaration of Helsinki. Written informed consent was obtained from all patients.

This study employed a retrospective design. Of 1881 consecutive patients (2257 hips) who underwent THA with a posterior approach at our institution between February 2003 and January 2018, 48 patients (48 hips) with a history of unilateral spontaneous hip arthrodesis were considered eligible for this study. No patients had surgical hip arthrodesis. The patients’ characteristics are shown in Table 1. The exclusion criteria were as follows: (1) a history of THA performed for any reason other than ankylosed hip (2,162 hips in 1,786 patients); (2) no history of hip arthrodesis (10 hips in 10 patients); (3) history of THA or bipolar hip arthroplasty or spine surgery (14 hips in 14 patients); and (4) the absence of evaluable data (23 hips in 23 patients).

**Table 1:** Patient characteristics.

Characteristics	(*n* = 48)
Gender (male/female)	12/36
Age at conversion THA* (years)	64.0 ± 7.6
Height (cm)	154.1 ± 9.3
Weight (kg)	56.0 ± 9.3
Body mass index* (kg/m^2^)	23.5 ± 2.8
Duration of arthrodesis* (years)	37.3 ± 16.0
Cause of hip arthrodesis	
Osteoarthritis, *n* (%)	16 (33.3)
Tuberculosis, *n* (%)	12 (25.0)
Fracture, *n* (%)	7 (14.6)
Infection, *n* (%)	3 (6.3)
NA, *n* (%)	10 (20.8)
Operative side (right/left)	21/27
Hip joint at non-operative side	
Normal, *n* (%)	29 (60.4)
Osteoarthritis, *n* (%)	19 (39.6)
Coronal lumbar-pelvic-femoral alignment	
Preoperative LS* (°)	10.8 ± 9.2
Preoperative PO** (°)	6.8 (3.9 to 10.0)
Preoperative HAA** (°)	10.0 (4.7 to 15.5)
Preoperative LLD (cm)	− 1.9 ± 2.8
Hip ROM	
Preoperative flexion* (°)	24.3 ± 16.9
Preoperative extension* (°)	− 13.8 ± 15.9
Preoperative abduction* (°)	4.6 ± 12.4
Preoperative adduction* (°)	2.2 ± 13.3
Preoperative external rotation* (°)	7.0 ± 10.1
Preoperative internal rotation** (°)	− 4.6 ± 10.3
JOA hip score	
Preoperative total score* (points)	56.7 ± 14.9
Preoperative pain** (points)	35.0 (20.0 to 40.0)
Preoperative range of motion** (points)	1.0 (0 to 4.0)
Preoperative ability to walk** (points)	15.0 (10.0 to 15.0)
Preoperative activity of daily life** (points)	12.0 (10.0 to 12.0)

*THA*, total hip arthroplasty. *ROM*, range of motion. *NA*, not available. *JOA*, Japanese Orthopaedic Association.

*Values are the mean ± standard deviation. **Values are the median (interquartile range).

### Patients’ demographics

We collected data concerning the gender, age at conversion THA, height, weight, body mass index, duration of arthrodesis, cause of hip arthrodesis, operative side, and hip joint on the non-operative side.

### Radiographic examination

**Figure 1:**
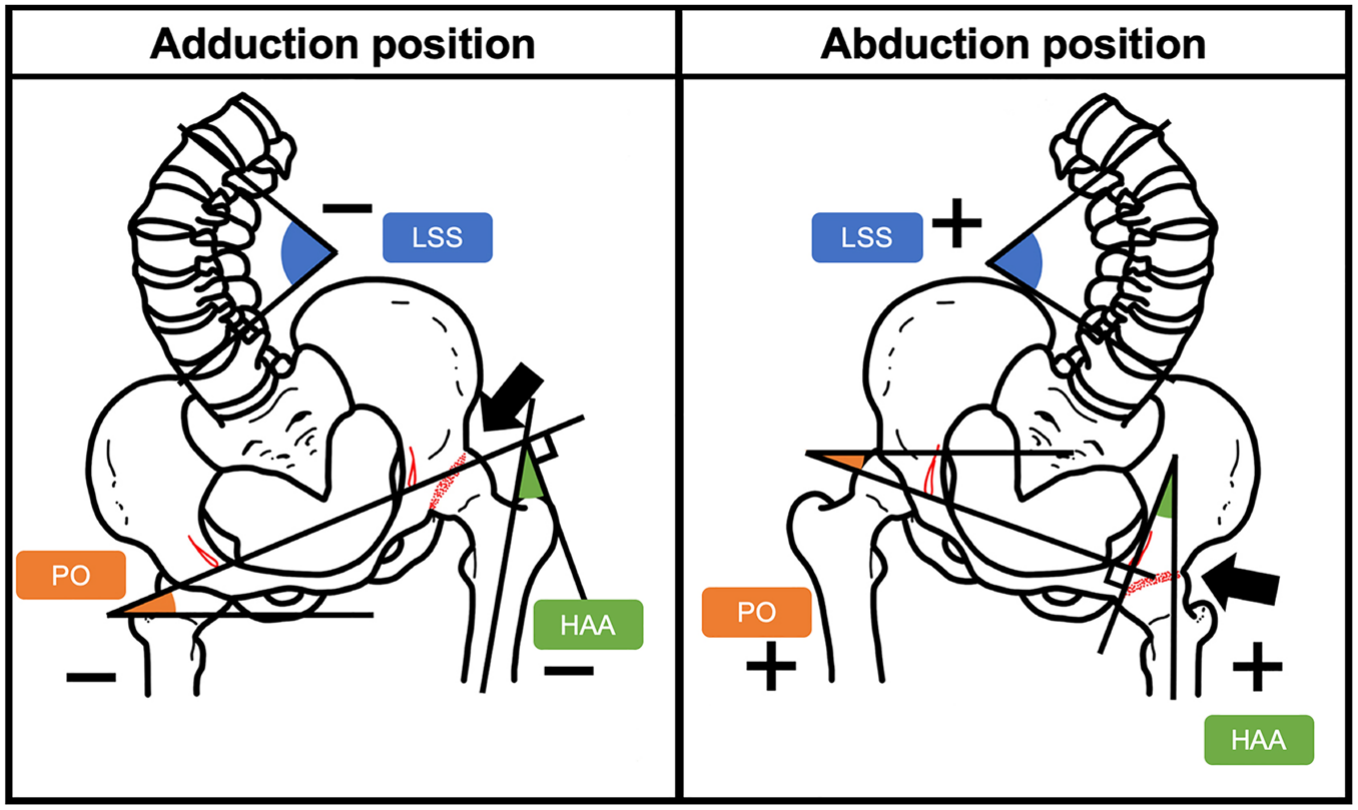
Illustration of a standing anterior–posterior radiograph showing adduction and abduction hip deformities on the left side. *LS*, lumbar scoliosis; *PO*, pelvic obliquity; *HAA*, hip adduction angle.

All patients underwent standing anterior–posterior spine-pelvis-hip radiographs before and at least 12 months after THA. On the radiographs, Cobb’s angle of lumbar scoliosis (LS), the pelvic obliquity (PO) angle, and the hip adduction angle (HAA) were measured (Fig. 1); the LS was measured using Cobb’s method at the apical level defined as the point of the most laterally deviated vertebra in a scoliosis curve^13,14^. This value was marked as positive in calculations when the convexity faced the fused hip side and negative when the convexity faced contra-laterally. The PO angle was measured as the angle between the horizontal line and the line passing through the inferior tip of the bilateral pelvic teardrops^13,14^. The angle was marked as positive in calculations when the downward direction was toward the fused hip side and negative when the downward direction was toward the opposite side. The HAA was defined as the angle between the perpendicular angle of the line passing through the inferior tip of the bilateral pelvic teardrops and the long axis of the femur on the affected side^15^. This value was calculated with abduction regarded as a positive angle and adduction regarded as a negative angle.

The differences of preoperative and postoperative LS, PO, and HAA were defined as delta (Δ) LS, ΔPO, and ΔHAA, respectively. Since the normal LS was reported to be 0°^13^, the LS was assessed as follows: improved (|ΔLS|> 5° and postoperative LS approached 0°), unchanged (|ΔLS|≤ 5°), and progressed (|ΔLS|> 5° and postoperative LS moved away from 0°)^13^. Since the normal PO was reported to be 0°^13^, the PO was assessed as follows: improved (|ΔPO|> 3.5° and postoperative PO approached 0°), unchanged (|PO|≤ 3.5°), and progressed (|ΔPO|> 3.5° and postoperative PO moved away from 0°)^13^. Since the normal HAA was reported to be  − 8°^16^, the HAA was assessed as follows: improved (|ΔHAA|> 3.5° and postoperative HAA approached  − 8°), unchanged (|ΔHAA|≤ 3.5°), and progressed (|ΔHAA|> 3.5° and postoperative HAA moved away from  − 8°)^13^.

### Physical examination

Leg length discrepancy (LLD) was defined by the spinomalleolus distance on the healthy side from the spinomalleolus distance on the diseased side. The spinomalleolus distance was defined as the distance from the anterior superior iliac spine to the medial malleolus using a measuring tape. The differences of preoperative and postoperative LLD were defined as ΔLLD. When the spinomalleolus distance on the diseased side was longer than that on the healthy side, it was defined as positive. Otherwise, it was defined as negative.

Passive hip range of motion (ROM) at operative side was assessed before THA. Hip flexion, abduction, adduction, internal rotation, and external rotation were measured in the supine position. Hip extension was measured in the prone position.

### Clinical outcomes

The clinical outcome was evaluated using the Japanese Orthopaedic Association (JOA) hip score^17^, which is a 100-point scale comprising the subcategories of pain (0–40 points), range of motion (0–20 points), ability to walk (0–20 points), and activities of daily living (0–20 points). The maximum total JOA score for a normal hip is 100 points, with higher scores indicating a better function. The JOA hip score was evaluated before and at least 12 months after THA. The difference in the preoperative and postoperative JOA hip score was defined as the ΔJOA hip score.

### Statistical analyses

Radiographic parameters (i.e., preoperative LS, PO, and HAA) were measured twice on separate days by two assessors (T.K. and T.Y.) and the intra-observer and inter-observer reliability were calculated. Values of < 0.40, 0.40–0.75, and > 0.75 are considered to indicate poor, good, and excellent reliability, respectively^18^.

The normality of distribution of quantitative data was determined using the Kolmogorov–Smirnov test. For normally distributed variables, the equality of variance between groups was tested using Levene’s test. A paired samples t-test was used to compare absolute values of LS and the JOA hip score between two related groups (i.e., preoperative vs. postoperative), as values that showed normal distribution with equal variance. The Wilcoxon signed-rank test was used for comparing absolute values of PO and HAA between two related groups (i.e., preoperative vs. postoperative), as values that did not show normal distribution. Student’s t-test was used to compare the absolute values of ΔPO and ΔHAA and ΔJOA hip score between two independent groups (i.e., normal vs. osteoarthritis at non-operative side), as values that showed normal distribution with equal variance. The Mann–Whitney's U test was used to compare the absolute value of ΔLS between two independent groups (i.e., normal vs. osteoarthritis at non-operative side), as values that did not show normal distribution.

Descriptive analyses were performed to assess the distribution of improved, unchanged, and progressed LS, PO, and HAA.

Pearson’s correlation coefficient (r) was calculated for variables with a normal distribution, and Spearman’s correlation coefficient (ρ) was calculated for variables without a normal distribution to assess the relationship between changes in alignment (i.e., ΔLS, ΔPO, and ΔHAA) and possible factors (i.e., age at conversion THA, body mass index, duration of arthrodesis, preoperative LS, PO, HAA, LLD, ΔLLD, passive hip ROM at operative side, and ΔJOA hip score). Correlation coefficients of 0.00–0.10, 0.10–0.39, 0.40–0.69, 0.70–0.89, and 0.90–1.00 were defined as negligible, weak, moderate, strong, and very strong correlation, respectively^19^.

P-values of < 0.05 were considered to indicate statistical significance. All analyses were performed using JMP® pro 14 (SAS Institute, Cary, North Carolina, USA).

### Ethical approval

All procedures performed in studies involving human participants were in accordance with the ethical standards of the institutional review board of Saga University Hospital (Registration No.: 2015-12-13) and with the 1964 Declaration of Helsinki and its later amendments or comparable ethical standards.

### Informed consent

Informed consent was obtained from all subjects.

## Results

### Intra-observer and inter-observer reliability

The estimated mean intra-observer reliability for the measurement of preoperative LS, PO, and HAA was 0.95, 0.95, and 0.96, respectively. The mean inter-observer reliability for the measurement of preoperative LS, PO, and HAA was 0.88, 0.90, and 0.91, respectively. The intra-observer and inter-observer reliability were therefore judged to be excellent.

### Changes of alignment after conversion THA

**Figure 2:**
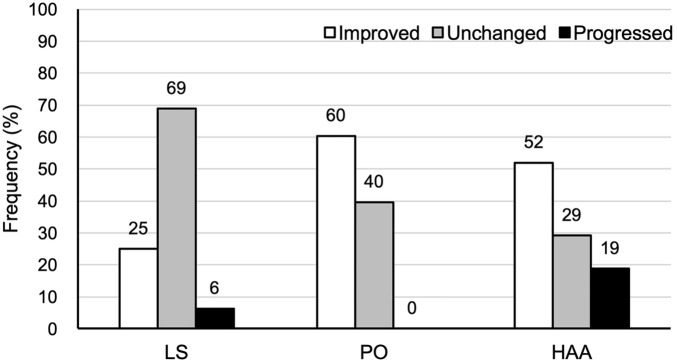
Distribution of the improved, unchanged, and progressed changes in the coronal lumbar-pelvic-femoral alignment. *LS*, lumbar scoliosis; *PO*, pelvic obliquity; *HAA*, hip adduction angle.

The comparison of preoperative and postoperative coronal alignment is shown in Table 2 and the distribution of the changes in coronal alignment is shown in Fig. 2. Preoperative LS was significantly higher than postoperative LS (mean, 10.8° vs. 8.2°, p = 0.004). Preoperative PO was significantly higher than postoperative PO (median, 6.8° vs. 2.0°, p < 0.001). The preoperative HAA was significantly higher than the postoperative HAA (median, 10.0° vs. 6.0°, p = 0.003).

**Table 2:** A comparison of the absolute values of preoperative and postoperative coronal lumbar-pelvic-femoral alignment and of the preoperative and postoperative JOA hip scores.

	Preoperative (*n* = 48)	Postoperative (*n* = 48)	*p* value
LS** (°)	9.0 (2.2 to 15.5)	5.5 (1.7 to 12.4)	0.005
PO** (°)	6.8 (3.9 to 10.0)	2.0 (0.9 to 4.0)	< 0.001
HAA** (°)	10.0 (4.7 to 15.5)	6.0 (3.0 to 13.6)	0.003
LLD* (cm)	− 1.9 ± 2.8	− 1.2 ± 2.2	0.159
JOA hip score* (points)	56.7 ± 14.9	81.2 ± 9.5	< 0.001

*LS*, lumbar scoliosis; *PO*, pelvic obliquity; *HAA*, hip adduction angle. *JOA*, Japanese Orthopaedic Association.

*Values are the mean ± standard deviation and were compared using a paired samples *t*-test. **Values are the median (interquartile range) and were compared using the Wilcoxon signed-rank test.

Of 48 patients, 12 (25.0%), 33 (68.8%) and 3 (6.3%) had improved, unchanged, and progressed LS, respectively. Of 48 patients, 29 (60.4%), 19 (39.6%) and 0 (0%) had improved, unchanged, and progressed PO, respectively. Of 48 patients, 25 (52.0%), 14 (29.2%) and 9 (18.8%) had improved, unchanged, and progressed HAA, respectively (Fig. 2).

### Factors associated with changes in alignment after conversion THA

Correlations between the change in the coronal lumbar-pelvic-femoral alignment after conversion THA and possible associated factors are shown in Table 3. ΔLS was correlated with the preoperative LS (ρ =  − 0.621, p < 0.001), preoperative PO (ρ =  − 0.580, p < 0.001), and preoperative HAA (ρ =  − 0.467, p < 0.001). ΔPO was correlated with the preoperative LS (ρ =  − 0.671, p < 0.001), preoperative PO (ρ =  − 0.892, p < 0.001), preoperative HAA (ρ =  − 0.728, p < 0.001), preoperative abduction (r =  − 0.348, p = 0.040), and preoperative adduction (r = 0.342, p = 0.049). ΔHAA was correlated with duration of arthrodesis (r =  − 0.735, p < 0.001), preoperative LS (ρ =  − 0.613, p < 0.001), preoperative PO (ρ =  − 0.751, p < 0.001), preoperative HAA (ρ =  − 0.824, p < 0.001), preoperative abduction (r =  − 0.350, p = 0.039), and preoperative adduction (r = 0.419, p = 0.123). ΔLS, ΔPO and ΔHAA were not significantly correlated with other factors, and there were no significant differences in the absolute values of ΔLS, ΔPO and ΔHAA between patients with normal and osteoarthritic hip joints on the non-operative side (Table 4).

**Table 3:** Pearson’s or Spearman’s correlation between the change in the coronal lumbar-pelvic-femoral alignment after conversion to THA and possible associated factors.

	ΔLS (°)	ΔPO (°)	ΔHAA (°)
Age at conversion THA (years)	0.029	− 0.157	− 0.139
Height (cm)	0.276	0.118	0.096
Weight (kg)	0.150	− 0.037	− 0.107
Body mass index (kg/m^2^)	− 0.136	− 0.170	− 0.244
Duration of arthrodesis (years)	− 0.028	− 0.180	− 0.735****
Preoperative LS (°)	− 0.621****	− 0.671****	− 0.613****
Preoperative PO (°)	− 0.581****	− 0.892****	− 0.751****
Preoperative HAA (°)	− 0.467****	− 0.728****	− 0.824****
Preoperative LLD (cm)	0.048	0.014	0.123
ΔLLD (cm)	0.059	0.020	0.028
Preoperative flexion (°)	− 0.014	− 0.032	− 0.044
Preoperative extension (°)	0.153	0.145	0.248
Preoperative abduction (°)	− 0.065	− 0.348*	− 0.350*
Preoperative adduction (°)	0.185	0.342*	0.419*
Preoperative external rotation (°)	0.279	− 0.150	− 0.193
Preoperative internal rotation (°)	− 0.256	0.036	0.133
ΔJOA hip score (points)	0.274	0.369*	0.399**

*THA*, total hip arthroplasty; *LS*, lumbar scoliosis; *PO*, pelvic obliquity; *HAA*, hip adduction angle; *JOA*, Japanese Orthopaedic Association.

*ΔLS*: the difference between the LS before and after surgery. *ΔPO*: difference between the PO before and after surgery. *ΔHAA*: difference between the HAA before and after surgery. *ΔJOA hip score*: difference between the JOA hip score before and after surgery.

Pearson’s correlation coefficient was calculated for variables without shade, and Spearman’s correlation coefficient was calculated for variables with shade. *p < 0.05, **p < 0.01, ***p < 0.005, ****p < 0.001.

**Table 4:** A comparison of the absolute value of the change in coronal lumbar-pelvic-femoral alignment after conversion THA and of the change in the JOA hip score after conversion THA stratified by hip joint on the non-operative side.

	Normal (*n* = 29)	Osteoarthritis (*n* = 19)	*p* value
ΔLS** (°)	2.6 (0.4 to 4.0)	3.6 (0.8 to 7.1)	0.384
ΔPO* (°)	5.6 ± 4.6	5.0 ± 3.9	0.639
ΔHAA* (°)	8.7 ± 7.8	9.6 ± 8.7	0.716
ΔJOA hip score* (points)	23.8 ± 14.3	25.4 ± 12.3	0.724

*LS*, lumbar scoliosis; *PO*, pelvic obliquity; *HAA*, hip adduction angle; *JOA*, Japanese Orthopaedic Association.

*ΔLS*: the difference between the LS before and after surgery. *ΔPO*: the difference between the PO before and after surgery. *ΔHAA*: the difference between the HAA before and after surgery. *ΔJOA hip score*: difference between the JOA hip score before and after surgery.

*Values are the mean ± standard deviation and were compared using Student’s *t*-test. **Values are the median (interquartile range) and were compared using the Mann–Whitney's *U* test.

### Clinical outcomes

The preoperative JOA hip score was significantly lower than the postoperative JOA hip score (mean, 56.7 points vs. 81.2 points, p < 0.001) (Table 2). The ΔJOA hip score was significantly correlated with ΔPO (r = 0.369, p = 0.015) and ΔHAA (r = 0.399, p = 0.008). In contrast. the ΔJOA hip score was not significantly correlated with ΔLS (ρ = 0.274, p = 0.076).

### Representative case

**Figure 3:**
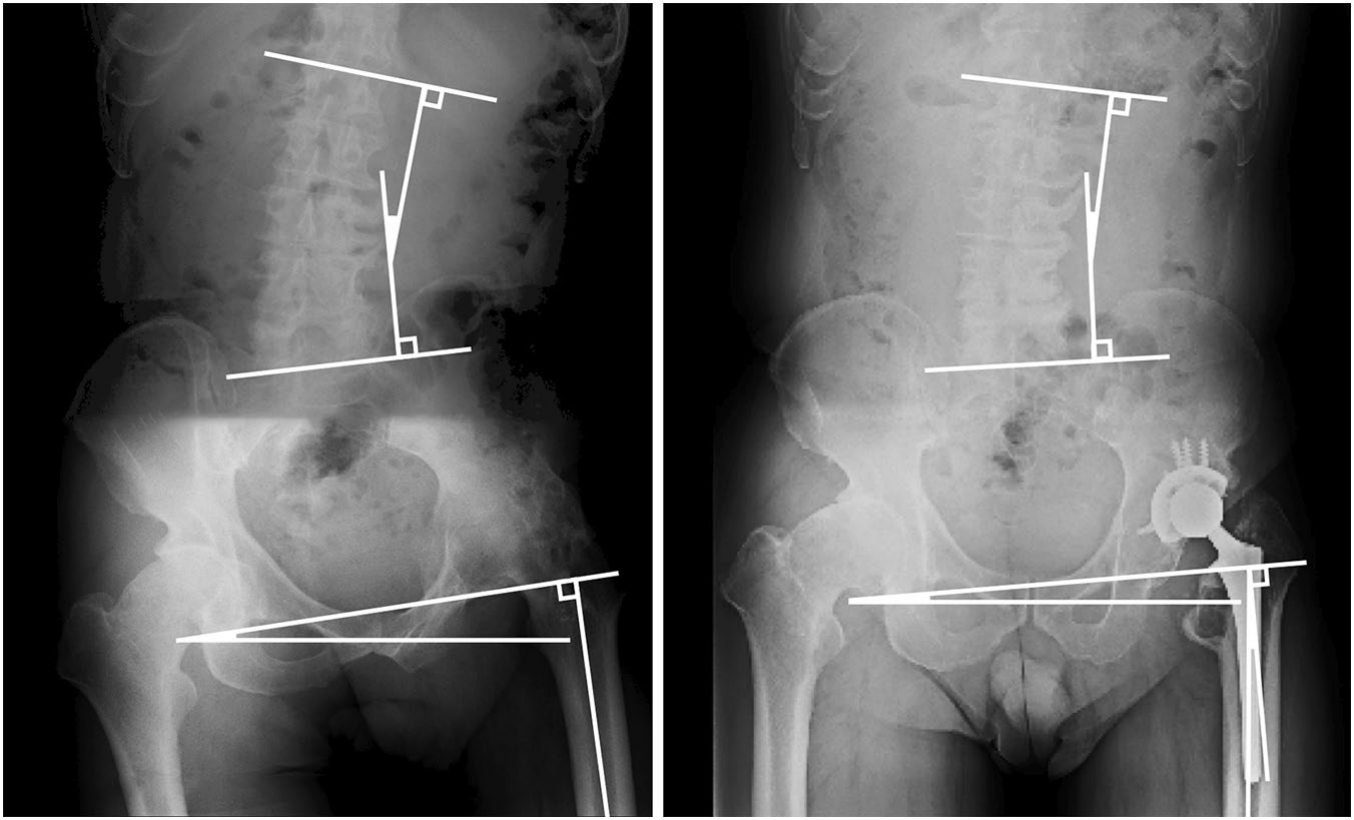
Preoperative and postoperative lumbo-pelvic radiographs. The preoperative LS, PO, HAA, and LLD were 44°, 7°, 0°, and  − 1 cm, respectively. The postoperative LS, PO, HAA, and LLD values were 37°, 2°, − 4°, 0 cm, respectively. *LS*, lumbar scoliosis; *PO*, pelvic obliquity; *HAA*, hip adduction angle; *LLD*, leg length discrepancy.

The patient was a 52-year-old man with a chief complaint of lumbago and hip pain, who had suffered from an ankylosed hip on adduction due to infection for 36 years. He underwent conversion THA (Fig. 3). The preoperative LS, PO, HAA, and LLD were 44°, 7°, 0°, and  − 1 cm, respectively. The postoperative LS, PO, HAA, and LLD values were 37°, 2°,  − 4°, 0 cm, respectively. Therefore, ΔLS, ΔPO, and ΔHAA were  − 7°,  − 5°, and  − 4°, respectively. Accordingly, LS was assessed as improved, since |ΔLS|> 5° and postoperative LS approached 0°; PO was assessed as improved, since |ΔPO|> 3.5° and postoperative PO approached 0°; and HAA was assessed as improved, since |ΔHAA|> 3.5° and postoperative HAA approached  − 8°. The preoperative and postoperative JOA hip score were 64 points and 90 points, respectively.

## Discussion

Our main findings were as follows: (1) LS, PO, and HAA were significantly improved due to conversion of hip arthrodesis to arthroplasty; and (2) preoperative coronal lumbar-pelvic-femoral alignment was the main determinant of improvement in LS, PO, and HAA after conversion THA.

Overall, we found a significant improvement in LS after conversion of hip arthrodesis to arthroplasty. To be more precise, 25% of patients showed improved LS. In addition, the larger improvement in LS was observed in patients with larger preoperative LS, PO, and HAA values. Contrarily, 6% of the patients showed progression in LS. The reported mechanisms of LS progression after conversion THA include de novo scoliosis with the rigid lumbosacral junction due to the long-term course or overcompensation at the lumbosacral junction and progression of L4 tilt^13,14,20–22^. A rigid spine can be detected on dynamic radiographs. This hypothesis should be investigated in future studies.

In this study, PO was significantly improved due to conversion of hip arthrodesis to arthroplasty. We found that 60% of patients showed improvement of PO and no patients showed progression of PO after conversion THA. Furthermore, a greater preoperative PO value was related to greater improvement in PO after conversion THA. It therefore seems likely that horizontalization of the pelvis is expected after conversion of hip arthrodesis to arthroplasty. These findings theoretically support the recommendation that acetabular inclination should be included in Lewinnek’s ‘safe zone’ (i.e., 40° ± 10°^23^) in patients with unilateral ankylosed hip^24^. However, postoperative mechanical failure (e.g., loosening and dislocation) and associated revision procedures are reported in 13–27% of patients^8–12^; thus further investigations regarding this issue is required.

We found that the HAA was significantly changed due to conversion of hip arthrodesis to arthroplasty. Notably, 19% of patients showed progression of the HAA after conversion THA. As shown in Table 3, ΔHAA was negatively correlated with the duration of hip arthrodesis. This indicates that contusion of hip adductor muscle may play an important role in ΔHAA. In addition, the shorter duration of arthrodesis was associated with the larger postoperative change in HAA after conversion THA. These findings may partially explain the relationship between shorter the duration of arthrodesis and higher mechanical failure rate; Peterson et al.^9^ reported that ≤ 30 years of arthrodesis was related to a higher failure rate in comparison to > 30 years of arthrodesis (40% vs. 6.7%, *p* < 0.05). Therefore, separation of the hip adductor muscle in addition to conversion THA should be considered, with reference to preoperative coronal lumbar-pelvic-femoral alignment and the duration of hip arthrodesis.

Unexpectedly, the main determinants of the postoperative change in PO and HAA were preoperative LS, PO, and HAA, and preoperative hip ROM for hip abduction and adduction, rather than preoperative LLD, ΔLLD, or the state of the contralateral hip joint. These findings may have been influenced by our study methods; spinomalleolus distance measurements were found to show low reliability in comparison to radiological methods^24^. In addition, we did not assess the severity of hip osteoarthritis at the contralateral hip joint. Although we did not assess changes in hip ROM after conversion THA in this study, a movable hip joint after THA for abduction and adduction may have a great influence on high PO and HAA values in the standing position^25^.

Importantly, we first reported the relationship between changes in the coronal spino-pelvic alignment and in the clinical outcomes after conversion THA. The JOA hip score was significantly improved due to conversion of hip arthrodesis to arthroplasty. Furthermore, improvement in PO and HAA after conversion THA was the main determinant of improvement in the JOA hip score. In contrast, improvement in LS after conversion THA was not a significant determinant of improvement in the JOA hip score, possibly due to the fact that the JOA hip score mainly includes questionnaire items regarding hip. A gait analysis may be useful for assessing these relationships in detail.

### Limitations

The present study was associated with some limitations. First, the study population was limited to 48 patients. This is because the ideal candidates for hip arthrodesis are young adults of < 40 years of age with symptomatic non-inflammatory monoarticular end-stage osteoarthritis and hip arthrodesis is an uncommon procedure in modern times because of the increased popularity of THA^2^. When the rarity of hip arthrodesis was taken into consideration, the sample size could be accepted as a strength of this study. Second, sagittal lumbar-pelvic-femoral alignment and internal/external rotation of the spine, pelvis, and femurs was not assessed in this study, which may have affected our results. For instance, improvement in the pelvis rotation can lead to radiographic PO improvement, as the radiographic position of tear drops can change in the rotated pelvis. Although the inter- and intra-observer reliabilities were excellent, the accuracy of the measurement method would have been limited. Third, there was a lack of unity in the period of the assessment of radiographs after THA. It is reported that time may affect the radiographic assessment after THA^26^; measurements at intervals of a few months can demonstrate dynamic changes in LS, PO, and HAA for evaluating the presence of continuous improvement. Although at least a 12-month follow-up was done in all cases, this limitation may have influenced our results. Finally, and most importantly, full-length standing radiographs for the spine and lower limbs as well as lumbar bending radiographs were not evaluated in this study. These images are strongly needed to make a precise evaluation of the coronal deformity of the lumbo-pelvic complex both pre- and post-operation, especially for patients with LLD. Further investigations regarding these issues are required.

## Conclusions

Significant improvements of the LS, PO, and HAA were observed due to conversion of hip arthrodesis to arthroplasty. A larger improvement in LS, PO, and HAA can be expected in patients with larger preoperative LS, PO, and HAA values. However, there may be some exceptions, and further investigations are necessary.

## Data Availability

The datasets used during the current study are not publicly available because of patient confidentiality but are available from the corresponding author on reasonable request.
